# Retraction: Association study of inflammatory cytokine and chemokine expression in hand foot and mouth disease

**DOI:** 10.18632/oncotarget.28655

**Published:** 2024-09-30

**Authors:** Wenzhong Shang, Suying Qian, Lijuan Fang, Yong Han, Cuiping Zheng

**Affiliations:** ^1^People’s Hospital of Fuyang District, Hangzhou, Zhejiang, P. R. China; ^2^The Second Hospital of Ningbo, Ningbo, Zhejiang, P. R. China; ^3^The Fourth Hospital of Xiaoshan District, Hangzhou, Zhejiang, P. R. China; ^4^Key Laboratory of Tumor Molecular Diagnosis and Individualized Medicine of Zhejiang Province, Zhejiang Provincial People’s Hospital, Hangzhou, Zhejiang, P. R. China; ^5^Dingli Hospital of Wenzhou Medical School, Wezhou Central Hospital, Zhejiang, P. R. China; ^*^These authors contributed equally to this work

**This article has been retracted:** Dr. Yong Han, one of the corresponding authors, contacted Oncotarget saying that their collaborators had already published the data presented in the Oncotarget paper in another paper/journal. He said “Since the main part of the data and conclusion were the same, this was a kind of duplicate publication. Although it was unintentional, after discussion with my co-authors, we decided to retract our paper.”

The Journal contacted the Chairman of the Science and Research Department at Zhejiang Provincial People’s Hospital, Professor Xianmin Tong, with an investigation request. The investigation confirmed a retraction. Therefore, with the agreement of all authors, the Editorial decision was made to retract the paper.

Original article: Oncotarget. 2017; 8:79425–79432. 79425-79432. https://doi.org/10.18632/oncotarget.18341

